# The burden of congenital birth defects between 1990 and 2019 in China: an observational study

**DOI:** 10.3389/fped.2023.1170755

**Published:** 2023-05-12

**Authors:** Yajun Zhao, Haonan Zhang, Minghui Peng, Yemei Zhou, Xuelin Cheng, Shijia Yang, Zhaoyu Zhang, Ming Liu, Xiaopan Li, Sunfang Jiang

**Affiliations:** ^1^ Department of General Practice, Zhongshan Hospital, Fudan University, Shanghai, China; ^2^ Department of Health Management Centre, Zhongshan Hospital, Fudan University, Shanghai, China; ^3^ Shanghai Engineering Research Centre of AI Technology for Cardiopulmonary Diseases, Zhongshan Hospital, Fudan University, Shanghai, China

**Keywords:** congenital birth defects, trend analysis, burden of disease, disability-adjusted life-years, China

## Abstract

**Background:**

Congenital birth defects (CBDs) are a major public health issue. This study aims to assess trends in the burden of CBDs between 1990 and 2019 across China based on the Global Burden of Disease Study 2019 (GBD 2019).

**Methods:**

Indicators of the burden of CBDs included incidence, mortality, and disability-adjusted life years (DALYs). Metrics included number, rate, and age-standardized rate with 95% uncertainty intervals (UIs). Data were stratified by region [China, global, high-, middle-, low-socio-demographic index (SDI)], age, sex, and type of CBD. Average annual percentage changes (AAPC) and trends were evaluated.

**Results:**

In China, between 1990 and 2019, the age-standardized incidence rate for CBDs showed an increasing trend, with an AAPC of 0.26% (0.11% to 0.41%), reaching 148.12 per 10^5^ person-years (124.03 to 176.33) in 2019. Most CBDs were congenital heart anomalies, with an AAPC of 0.12% (−0.08% to 0.32%). The age-standardized mortality rate for CBDs showed a decreasing trend, with an AAPC of −4.57% (−4.97% to −4.17%), reaching 4.62 per 10^5^ person-years (3.88 to 5.57) in 2019. Most mortality was associated with congenital heart anomalies, with an AAPC of −3.77% (−4.35% to −3.19%). The age-standardized DALYs rate for CBDs showed a decreasing trend, with an AAPC of −3.74% (−3.95% to −3.52%), reaching 480.95 per 10^5^ person-years (407.69 to 570.04) in 2019.

**Conclusions:**

Morbidity associated with CBDs increased in China between 1990 and 2019, accelerated by the adoption of the two-child policy, and ranked high globally. These findings emphasize the need for prenatal screening and primary and secondary prevention strategies.

## 1. Introduction

Congenital birth defects (CBDs) are structural and/or functional abnormalities that are present at birth. CBDs can develop at any stage of pregnancy, but most occur within the first three months of pregnancy. Globally, CBDs are the leading cause of infant mortality in the world (1, 2), with an estimated 3%–6% of infants, or nearly 8 million babies, born with severe birth defects each year (3). The true number of cases is likely higher, as terminations and stillbirths are not considered in these statistics. CBDs have become a major public health issue, leading to long-term disability and greatly impacting individuals, families, healthcare systems, and society.

Infant mortality is an important marker of the health of a society (4). The Sustainable Development Goals (SDGs) set by the World Health Organization (WHO) include a call to end, by 2030, preventable deaths of newborn babies, with all countries aiming to reduce infant mortality to at least as low as 12 per 1,000 live births (5). In China, tens of millions of infants are born every year (6, 7). Understanding the incidence and disease burden of CBDs in China will inform prevention and intervention measures against CBDs.

Most reports on the incidence and burden of CBDs are country-specific, and surveillance programs have been short (8, 9). In China, domestic reports on CBDs are relevant to a certain region or a specific congenital abnormality (10, 11). Long-term trends and the impact of recent changes in China's fertility policy on the occurrence of CBDs across China (12–15) are unknown. The objective of this study was to assess trends in the incidence and burden of CBDs between 1990 and 2019 across China based on the Global Burden of Disease Study 2019 (GBD 2019).

## 2. Methods

### 2.1. Data sources

GBD2019 data are accessible through a portal at the Institute for Health Metrics and Evaluation (IHME), University of Washington, United States. The GBD2019 dataset (https://ghdx.healthdata.org/gbd-2019) used modeling to estimate the burden of 369 diseases or injuries for 204 countries and territories worldwide between 1990 and 2019 (16, 17), stratified by factors such as cause, location, age, and sex.

In China, GBD2019 comprehensively estimated and evaluated the births and deaths of newborns in 31 provinces, autonomous regions, and municipalities, as well as the Hong Kong Special Administrative Region of China and the Macao Special Administrative Region of China (excluding Taiwan, China), using multiple data sources and adopting standardized and comparable methods. Data were mainly obtained from the China Health Statistics Yearbook, the China National Maternal and Child Health Surveillance System, China Vital Registration Birth Data, China Vital Registration Live Births Data, and some published literature or reports (16, 18).

### 2.2. Data extraction

The WHO attributes International Classification of Diseases (ICD), 10th version (https://icd.who.int/browse10/2010/en#/XVII) codes Q00-Q99 to eleven congenital malformations, deformations, and chromosomal abnormalities. Of these, data for neural tube defects, congenital heart anomalies, orofacial clefts, Down syndrome, Turner syndrome, Klinefelter syndrome, other chromosomal abnormalities, congenital musculoskeletal and limb anomalies, congenital urological anomalies, congenital anomalies of the digestive system, and other CBDs were available from the GBD.

Indicators of the burden of these CBDs, including incidence, mortality, and disability-adjusted life years (DALYs) were recorded. Metrics included number, rate, and age-standardized rate with 95% uncertainty intervals (UIs). Data were stratified by region [China, global, high-, middle-, low- socio-demographic index (SDI)], age, sex, and type of CBD. The SDI is a comprehensive indicator of a country's lag-distributed income per capita, average years of schooling, and the fertility rate in females under the age of 25 years (19). The data from China included in global, and China is classified as a High-middle SDI according to the SDI calculation rules. Age was categorized as 0–6 days, 7–27 days, 28–364 days, 1–4 years, 5–9 years, 10–14 years, 15–19 years, 20–54 years, and 55+ years after birth.

### 2.3. Statistical analysis

Data were sorted and analyzed using Excel 2021 software. The rate of change in incidence, mortality, and DALYs for CBDs between 1990 and 2019 was calculated as (2019 indicator value—1990 indicator value)/1990 indicator value × 100%. Trends in changes in incidence, mortality, and DALYs for CBDs between 1990 and 2019 were analyzed using Joinpoint Regression Program 4.7.0.0 software developed by the American Institute for Cancer Research (20). The number and location of segmentation points and corresponding *P* values were determined by the Monte Carlo permutation test, and the annual percentage change (APC) and average annual percentage change (AAPC) were calculated using log-linear regression. The incidence rate of CBDs between 2020 and 2030 in China was predicted using R-4.2.2 software. *P *< 0.05 was considered statistically significant.

## 3. Results

### 3.1. Incidence, mortality, and DALYs for CBDs stratified by region

**Figure 1:**
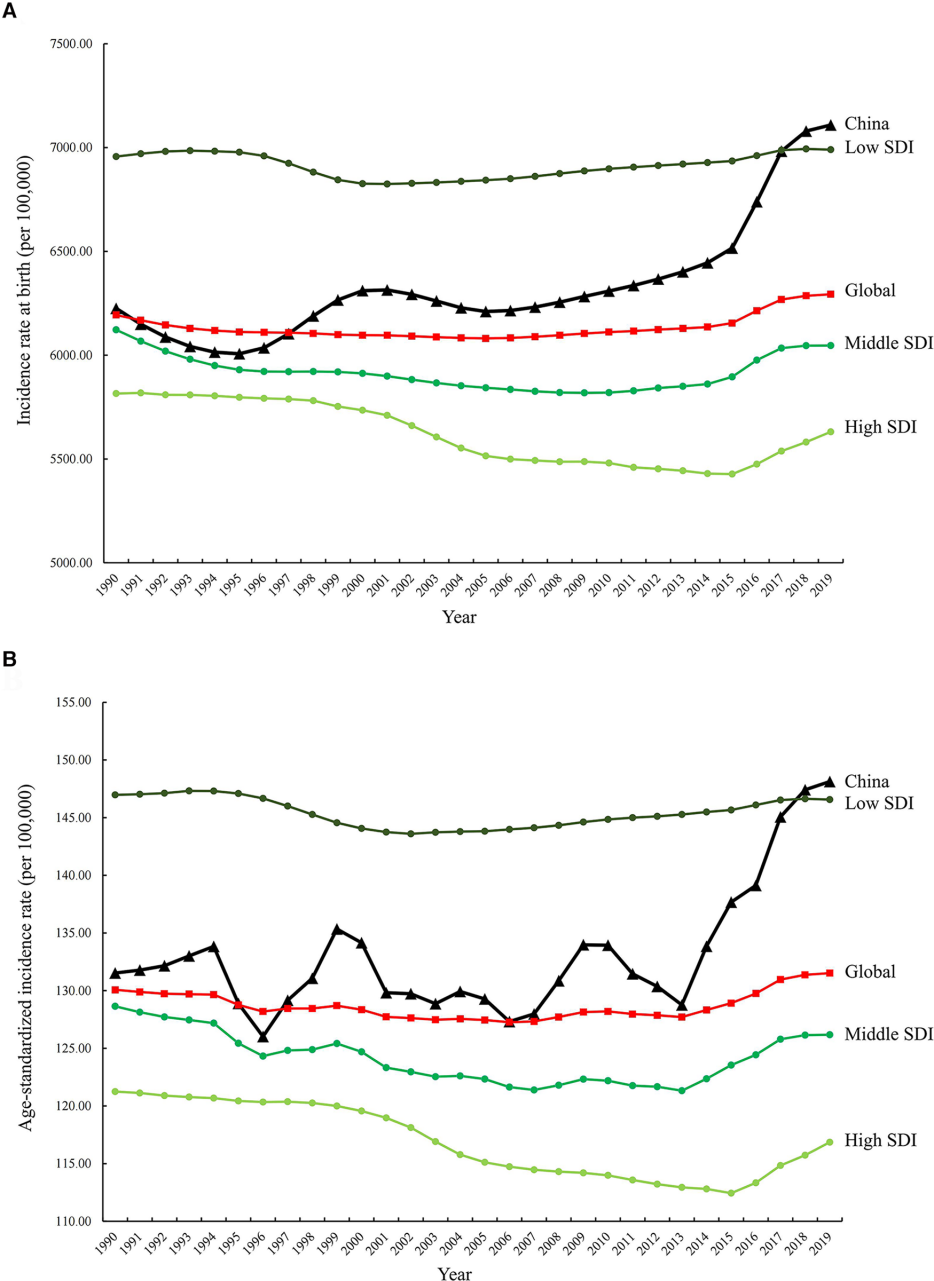
Incidence rates at birth (**A**) and age-standardized incidence rates (**B**) (per 100,000) for CBDs between 1990 and 2019 in China, globally, and high-, middle-, and low-SDI regions. *SDI: Socio-Demographic Index*.

**Figure 2:**
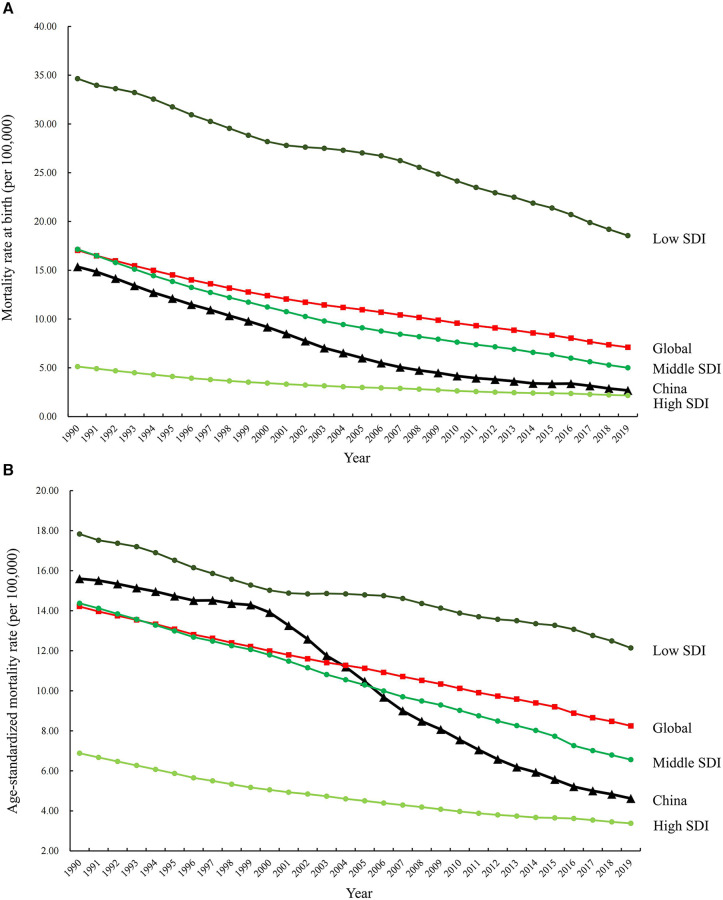
Mortality rates at birth (**A**) and age-standardized mortality rates (**B**) (per 100,000) for CBDs between 1990 and 2019 in China, globally, and high-, middle-, and low-SDI regions. *SDI: Socio-Demographic Index*.

**Figure 3:**
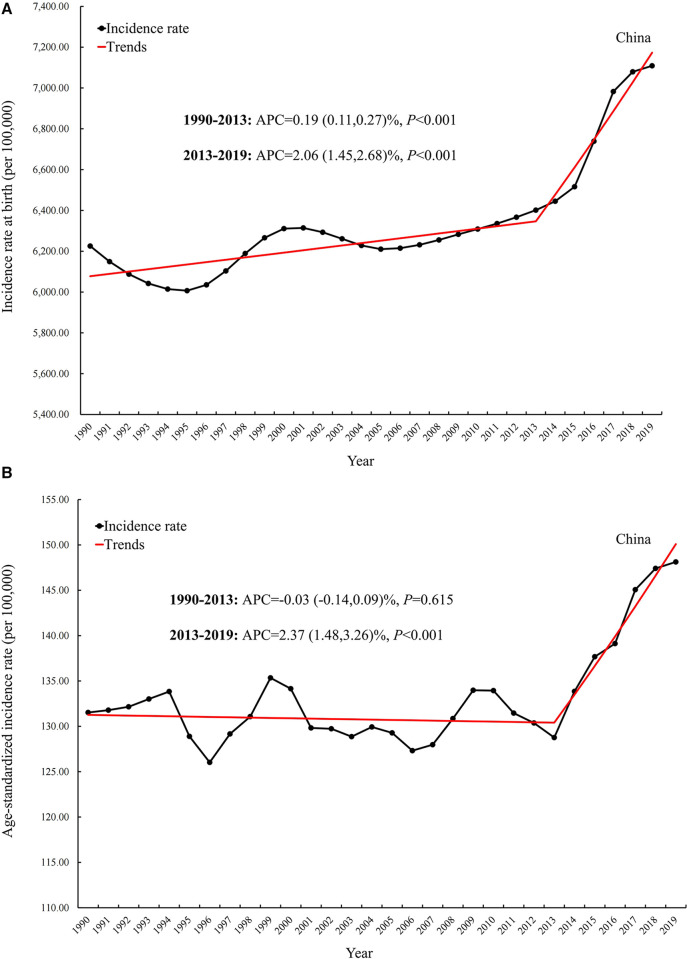
Incidence rate and trend at birth (**A**) and age-standardized incidence rate and trend (**B**) for CBDs (per 100,000) between 1990 and 2019 in China*. APC: Annual percentage change*.

**Figure 4:**
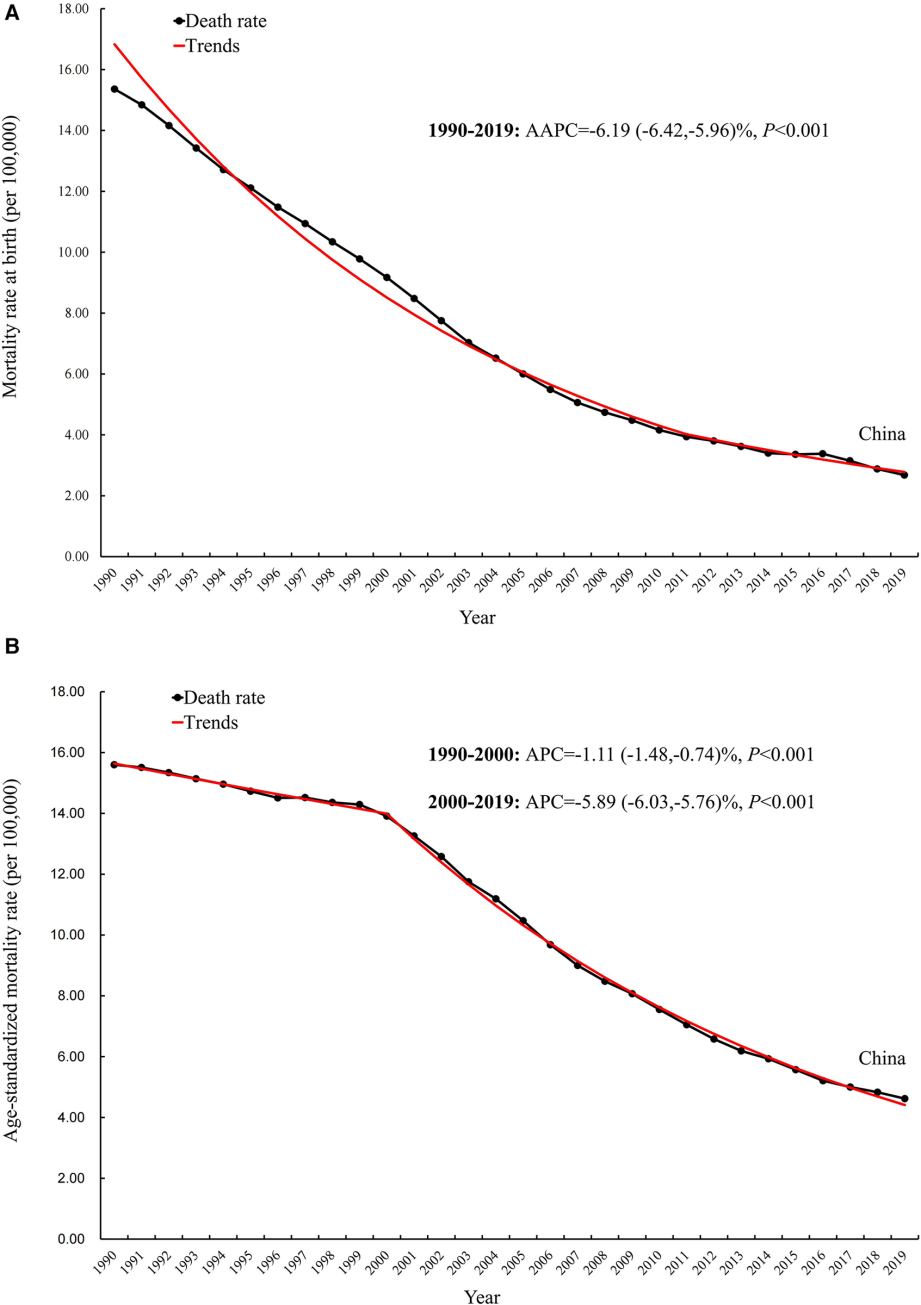
Mortality rate and trend at birth (**A**) and age-standardized mortality rate and trend (**B**) for CBDs (per 100,000) between 1990 and 2019 in China*. AAPC, average annual percentage changes; APC: Annual percentage change*.

**Figure 5:**
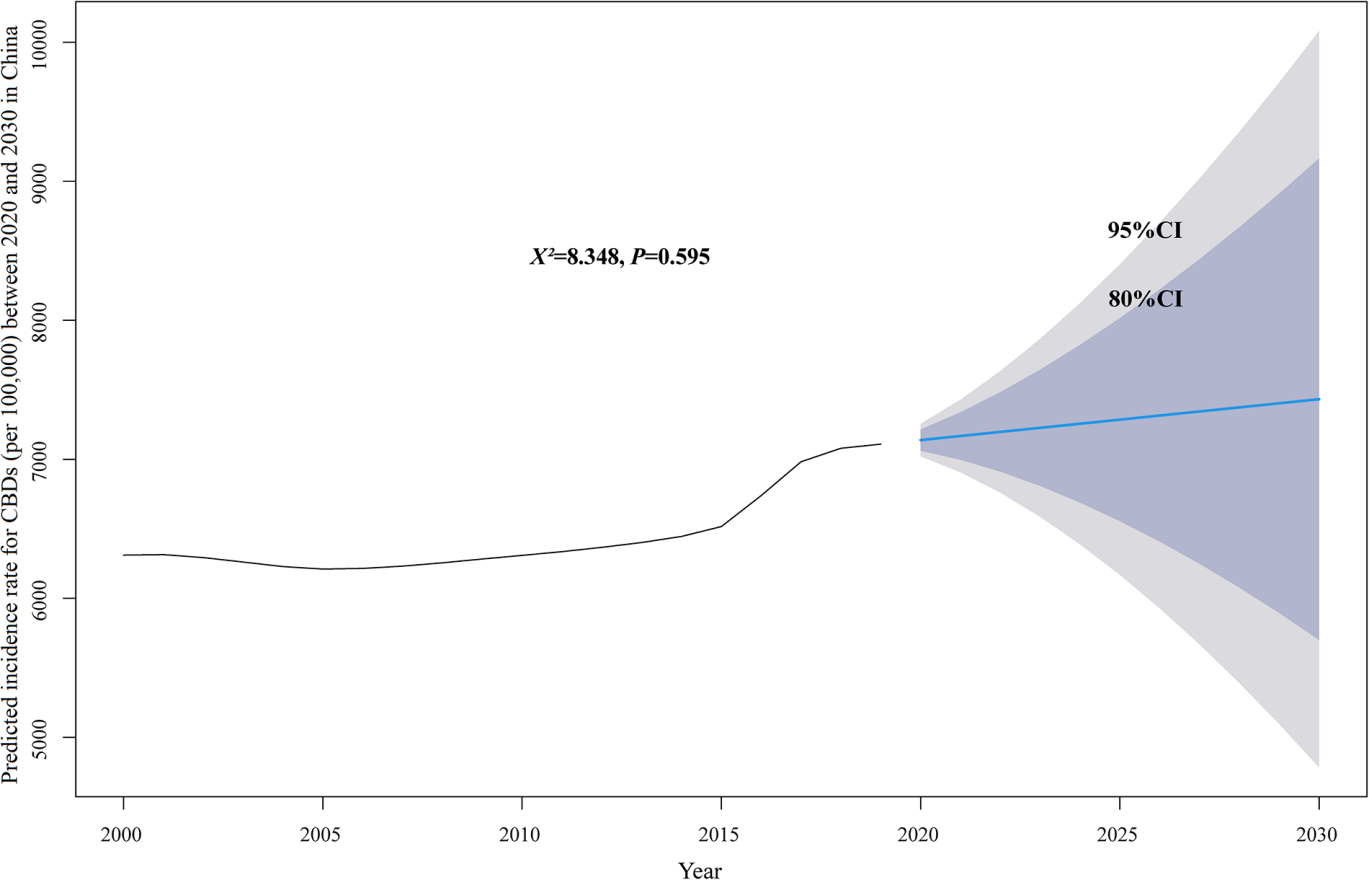
Predicted incidence rate for CBDs (per 100,000) between 2020 and 2030 in China. *CI, confidence interval*.

**Figure 6:**
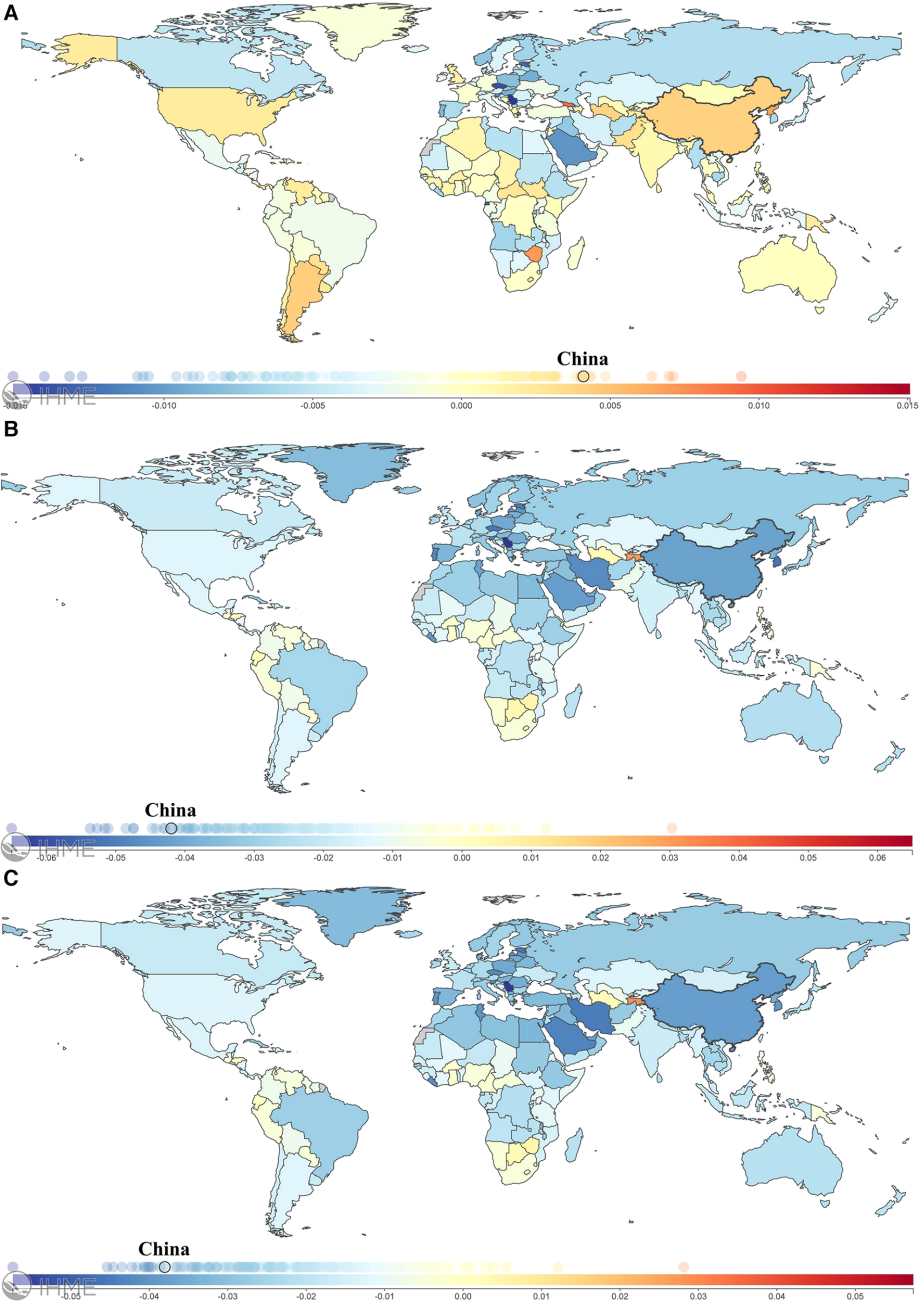
A world map of the changes in age-standardized incidence (**A**), mortality (**B**), and DALY (**C**) rates for CBDs between 1990 and 2019. *DALYs, Disability-Adjusted Life Years*. Figures from the Institute for Health Metrics and Evaluation (IHME), an independent global health research center at the University of Washington (cited from: https://vizhub.healthdata.org/gbd-compare/).

The incidence, mortality, and disability-adjusted life years (DALYs) for CBDs between 1990 and 2019 in China, globally, and high-, middle-, and low-SDI regions, expressed as numbers, rates, and age-standardized rates, are summarized in Tables 1–3, respectively. The AAPC in the age-standardized incidence rate for CBDs between 1990 and 2019 was 0.26% (95% CI: 0.11% to 0.41%) in China, 0.01% (95% CI: −0.03% to 0.05%) globally, −0.27% (95% CI: −0.32% to −0.21%) in high SDI regions, −0.11% (95% CI: −0.17% to −0.04%) in middle SDI regions, and −0.02% (95% CI: −0.06% to 0.01%) in low SDI regions (Table 1). The AAPC in the age-standardized mortality rate for CBDs between 1990 and 2019 was −4.57% (95% CI: −4.97% to −4.17%) in China, −1.79% (95% CI: −1.84% to −1.74%) globally, −2.40% (95% CI: −2.50% to −2.30%) in high SDI regions, −2.62% (95% CI: −2.74% to −2.51%) in middle SDI regions, and −1.16% (95% CI: −1.23% to −1.09%) in low SDI regions (Table 2). The AAPC in the age-standardized DALY rate for CBDs between 1990 and 2019 was −3.74% (95% CI: −3.95% to −3.52%) in China, −1.75% (95% CI: −1.82% to −1.69%) globally, −2.16% (95% CI: −2.22% to −2.10%) in high SDI regions, −2.49% (95% CI: −2.59% to −2.39%) in middle SDI regions, and −1.25% (95% CI: −1.31% to −1.19%) in low SDI regions (Table 3). Incidence and mortality rates at birth and age-standardized incidence and mortality rates (per 100,000) for CBDs between 1990 and 2019 in China, globally, and high-, middle-, and low-SDI regions are shown in Figures 1, 2. Incidence and mortality rates at birth, age-standardized incidence and mortality rates, and trends for CBDs (per 100,000) between 1990 and 2019 in China are shown in Figures 3, 4. The predicted incidence rate for CBDs (per 100,000) between 2020 and 2030 in China is shown in Figure 5. A world map of the changes in age-standardized incidence, mortality, and DALY rates for CBDs between 1990 and 2019 is shown in Figure 6. The age-standardized incidence rate of CBDs (%) between 1990 and 2019 in China, globally, and in India is shown in Supplementary Figure S1.

**Table 1:** Incidence for CBDs between 1990 and 2019 in China, globally, and high-, middle-, and low-SDI regions, expressed as numbers, rates, and age-standardized rates.

Categories	1990			2019			1990–2019	*P*
	Incidence number (95%UI)	Incidence rate (95%UI)	Age-standardized incidence rate (95%UI)	Incidence number (95%UI)	Incidence rate (95%UI)	Age-standardized incidence rate (95%UI)	Age-standardized incidence rate (AAPC 95%CI, %)	
China	15,15,941 (12,38,300, 18,22,313)	6,225.49 (5,085.31, 7,483.66)	131.52 (107.44, 158.11)	10,60,758 (888,247, 12,62,734)	7,108.76 (5,952.67, 8,462.32)	148.12 (124.03, 176.33)	0.26 (0.11, 0.41)	0.001
Global	85,55,498 (73,18,729, 99,62,971)	6,193.44 (5,298.13, 7,212.33)	130.07 (111.27, 151.47)	85,18,014 (73,04,958, 99,20,532)	6,293.32 (5,397.09, 7,329.54)	131.52 (112.79, 153.17)	0.01 (−0.03, 0.05)	0.634
High SDI	687,801 (595,622, 789,430)	5,815.41 (5,036.03, 6,674.68)	121.25 (105.00, 139.16)	579,904 (499,579, 667,625)	5,630.95 (4,850.98, 6,482.73)	116.86 (100.67, 134.54)	−0.27 (−0.32, −0.21)	<0.001
Middle SDI	26,58,409 (22,45,651, 31,33,131)	6,122.21 (5,171.64, 7,215.47)	128.65 (108.67, 151.62)	21,65,864 (18,47,983, 25,38,948)	6,046.58 (5,159.13, 7,088.14)	126.18 (107.66, 147.91)	−0.11 (−0.17, −0.04)	0.003
Low SDI	16,62,656 (14,05,787, 19,52,771)	6,956.80 (5,882.03, 8,170.69)	146.97 (124.27, 172.62)	26,46,268 (22,36,748, 31,14,322)	6,990.09 (5,908.35, 8,226.45)	146.56 (123.88, 172.49)	−0.02 (−0.06, 0.01)	0.208

AAPC, average annual percentage changes; UI, uncertainty interval; CI, confidence interval; SDI: Socio-Demographic Index.

**Table 2:** Mortality for CBDs between 1990 and 2019 in China, globally, and high-, middle-, and low-SDI regions, expressed as numbers, rates, and age-standardized rates.

Categories	1990			2019			1990–2019	*P*
	Mortality number (95%UI)	Mortality rate (95%UI)	Age-standardized mortality rate (95%UI)	Mortality number (95%UI)	Mortality rate (95%UI)	Age-standardized mortality rate (95%UI)	Age-standardized mortality rate (AAPC 95%CI,%)	
China	181,847 (142,817, 244,948)	15.36 (12.07, 20.69)	15.60 (12.22, 21.04)	38,049 (32,150, 45,255)	2.68 (2.26, 3.18)	4.62 (3.88, 5.57)	−4.57 (−4.97, −4.17)	<0.001
Global	911,943 (627,912, 13,41,147)	17.05 (11.74, 25.07)	14.21 (9.81, 20.87)	549,305 (447,797, 690,237)	7.10 (5.79, 8.92)	8.25 (6.71, 10.42)	−1.79 (−1.84, −1.74)	<0.001
High SDI	42,075 (38,816, 46,934)	5.12 (4.72, 5.71)	6.88 (6.33, 7.65)	21,908 (19,595, 24,737)	2.16 (1.93, 2.44)	3.38 (3.04, 3.91)	−2.40 (−2.50, −2.30)	<0.001
Middle SDI	294,343 (221,846, 419,955)	17.15 (12.92, 24.46)	14.37 (10.85, 20.47)	119,868 (99,465, 145,253)	5.00 (4.15, 6.06)	6.56 (5.40, 7.99)	−2.62 (−2.74, −2.51)	<0.001
Low SDI	182,936 (76,888, 334,890)	34.64 (14.56, 63.41)	17.83 (7.58, 32.62)	209,407 (126,038, 326,976)	18.55 (11.17, 28.97)	12.14 (7.43, 18.81)	−1.16 (−1.23, −1.09)	<0.001

AAPC, average annual percentage changes; UI, uncertainty interval; CI, confidence interval; SDI, Socio-Demographic Index.

**Table 3:** DALYs for CBDs between 1990 and 2019 in China, globally, and high-, middle-, and low-SDI regions, expressed as numbers, rates, and age-standardized rates.

Categories	1990			2019			1990–2019	*P*
	DALYs number (95%UI)	DALYs rate (95%UI)	Age-standardized DALYs rate (95%UI)	DALYs number (95%UI)	DALYs rate (95%UI)	Age-standardized DALYs rate (95%UI)	Age-standardized DALYs rate (AAPC 95%CI,%)	
China	16,91,1623 (13,40,2158, 22,59,0510)	1,428.73 (1,132.24, 1,908.49)	1,449.36 (1,147.93, 1,939.12)	42,15,525 (35,88,757, 50,40,152)	296.38 (252.31, 354.35)	480.95 (407.69, 570.04)	−3.74 (−3.95, −3.52)	<0.001
Global	84,13,6962 (59,23,5262, 12,19,86791)	1,572.7 (1,107.23, 2,280.19)	1,315.22 (927.28, 1,904.09)	52,78,4936 (43,60,1536, 65,52,2204)	682.2 (563.51, 846.82)	788.75 (650.07, 984.61)	−1.75 (−1.82, −1.69)	<0.001
High SDI	40,31,025 (36,90,164, 44,67,740)	490.38 (448.92, 543.51)	657.61 (604.98, 725.96)	22,16,653 (19,66,221, 25,21,364)	218.74 (194.03, 248.81)	349.79 (310.35, 400.16)	−2.16 (−2.22, −2.10)	<0.001
Middle SDI	27,28,2788 (20,81,9455, 38,64,0885)	1,589.21 (1,212.72, 2,250.81)	1,336.99 (1,020.59, 1,888.05)	11,96,6034 (10,03,6741, 14,28,4445)	499.3 (418.8, 596.04)	643.38 (537.37, 773.97)	−2.49 (−2.59, −2.39)	<0.001
Low SDI	16,65,7957 (73,47,902, 30,11,4181)	3,154.08 (1,391.28, 5,701.93)	1,641.19 (739.24, 2,960.19)	19,45,7068 (12,11,7048, 29,77,5061)	1,723.88 (1,073.56, 2,638.05)	1,135.13 (717.88, 1,724.04)	−1.25 (−1.31, −1.19)	<0.001

DALYs, Disability-Adjusted Life Years; SDI, Socio-Demographic Index; AAPC, average annual percentage changes; UI, uncertainty interval; CI, confidence interval.

### 3.2. Incidence, mortality, and DALYs for CBDs stratified by sex and type of CBD

The incidence and mortality for CBDs between 1990 and 2019 in China stratified by sex and type of CBD, expressed as numbers, rates, and age-standardized rates, are summarized in Tables 4, 5. The AAPC in the age-standardized incidence rate for CBDs between 1990 and 2019 was 0.28% (95% CI: 0.12% to 0.44%) in males and 0.12% (95% CI: −0.08% to 0.32%) for congenital heart anomalies (Table 4). The AAPC in the age-standardized mortality rate for CBDs between 1990 and 2019 was −4.49% (95% CI: −4.87% to −4.12%) in males and −3.77% (95% CI: −4.35% to −3.19%) for congenital heart anomalies (Table 5). The incidence and mortality for each type of CBD between 1990 and 2019 expressed as numbers, rates, and trends in China are summarized in Supplementary Tables S1, S2.

**Table 4:** Incidence for CBDs between 1990 and 2019 in China stratified by sex and type of CBD, expressed as numbers, rates, and age-standardized rates.

Categories	1990			2019			1990–2019	*P*
	Incidence number (95%UI)	Incidence rate (95%UI)	Age-standardized incidence rate (95%UI)	Incidence number (95%UI)	Incidence rate (95%UI)	Age-standardized incidence rate (95%UI)	Age-standardized incidence rate (AAPC 95%CI,%)	
Gender								
Male	834,890 (692,344, 10,00,789)	136.83 (113.46, 164.01)	136.53 (113.22, 163.66)	5,90,917 (4,95,136, 7,00,839)	81.53 (68.31, 96.69)	153.15 (128.33, 181.64)	0.28 (0.12, 0.44)	0.002
Female	6,81,051 (5,47,934, 8,28,914)	118.75 (95.54, 144.54)	125.87 (101.26, 153.19)	4,69,841 (3,91,027, 5,61,867)	67.36 (56.06, 80.55)	142.25 (118.39, 170.11)	0.22 (0.09, 0.36)	0.003
Top 5 congenital birth defects								
Congenital heart anomalies	5,79,009 (4,29,923, 7,79,088)	48.92 (36.32,65.82)	50.24 (37.30, 67.59)	3,50,912 (2,56,923, 4,85,179)	24.67 (18.06, 34.11)	49.00 (35.88, 67.75)	0.12 (−0.08, 0.32)	0.219
Congenital musculoskeletal and limb anomalies	5,43,511 (3,29,192, 7,87,795)	45.92 (27.81, 66.55)	47.16 (28.56, 68.35)	3,40,693 (2,19,348, 4,76,402)	23.95 (15.42, 33.49)	47.57 (30.63, 66.52)	−0.11 (−0.21, −0.02)	0.024
Digestive congenital anomalies	86,782 (67,636, 1,09,452)	7.33 (5.71, 9.25)	7.53 (5.87, 9.50)	1,76,411 (1,28,963, 2,29,517)	12.40 (9.07, 16.14)	24.63 (18.01, 32.05)	4.26 (2.73, 5.82)	<0.001
Urogenital congenital anomalies	1,27,137 (88,589, 1,77,239)	10.74 (7.48, 14.97)	11.03 (7.69, 15.38)	92,955 (64,038, 1,30,612)	6.54 (4.50, 9.18)	12.98 (8.94, 18.24)	−0.19 (−0.80, 0.43)	0.538
Other chromosomal abnormalities	1,04,814 (85,172, 1,27,120)	8.85 (7.20, 10.74)	9.09 (7.39, 11.03)	66,689 (53,178, 83,049)	4.69 (3.74, 5.84)	9.31 (7.43, 11.60)	−0.03 (−0.16, 0.10)	0.651

AAPC, average annual percentage changes; UI, uncertainty interval; CI, confidence interval.

**Table 5:** Mortality for CBDs between 1990 and 2019 in China stratified by sex and type of CBD, expressed as numbers, rates, and age-standardized rates.

Categories	1990			2019			1990–2019	*P*
	Mortality number (95%UI)	Mortality rate (95%UI)	Age-standardized mortality rate (95%UI)	Mortality number (95%UI)	Mortality rate (95%UI)	Age-standardized mortality rate (95%UI)	Age-standardized mortality rate (AAPC 95%CI,%)	
Gender								
Male	1,01,892 (64,864, 1,45,445)	16.70 (10.63, 23.84)	16.54 (10.49, 23.62)	21,779 (18,213, 28,201)	3.00 (2.51, 3.89)	4.95 (4.10, 6.55)	−4.49 (−4.87, −4.12)	<0.001
Female	79,955 (59,980, 1,17,817)	13.94 (10.46, 20.54)	14.54 (10.90, 21.48)	16,271 (13,596, 19,669)	2.33 (1.95, 2.82)	4.24 (3.53, 5.16)	−4.68 (−5.12, −4.24)	<0.001
Top 5 congenital birth defects								
Congenital heart anomalies	99,568 (77,975, 1,35,785)	8.41 (6.59, 11.47)	8.51 (6.66, 11.63)	25,312 (21,314, 30,078)	1.78 (1.50, 2.11)	3.01 (2.49, 3.63)	−3.77 (−4.35, −3.19)	<0.001
Congenital musculoskeletal and limb anomalies	1,535 (994, 2373)	0.13 (0.08, 0.20)	0.13 (0.08, 0.20)	277 (222, 380)	0.02 (0.02, 0.03)	0.03 (0.02, 0.04)	−6.14 (−6.85, −5.42)	<0.001
Digestive congenital anomalies	16,851 (7541, 28905)	1.42 (0.64, 2.44)	1.46 (0.65, 2.50)	3,308 (2214, 4440)	0.23 (0.16, 0.31)	0.44 (0.29, 0.59)	−4.42 (−4.60, −4.25)	<0.001
Urogenital congenital anomalies	1,498 (789, 2208)	0.13 (0.07, 0.19)	0.13 (0.07, 0.19)	346 (274, 421)	0.02 (0.02, 0.03)	0.04 (0.03, 0.05)	−4.96 (−5.31, −4.61)	<0.001
Other chromosomal abnormalities	803 (464, 1746)	0.07 (0.04, 0.15)	0.07 (0.04, 0.15)	507 (373, 689)	0.04 (0.03, 0.05)	0.06 (0.04, 0.08)	−0.07 (−0.36, 0.22)	0.615

AAPC, average annual percentage changes; UI, uncertainty interval; CI, confidence interval.

### 3.3. Age distribution of mortality for CBDs between 1990 and 2019 in China and globally

The mortality for CBDs between 1990 and 2019 in China and globally stratified by age (0–6 days, 7–27 days, 0–28 days) expressed as numbers, rates, and trends are summarized in Table 6. In China, the AAPC in the mortality rate at 0–6 days between 1990 and 2019 was −3.35% (−3.67% to −3.02%). In 2019, the number of deaths at 0–6 days was 7,253, and the number of deaths at 0–28 days was 10,602. Globally, the AAPC in the mortality rate at 0–6 days between 1990 and 2019 was −1.30% (−1.37% to −1.24%). In 2019, the number of deaths at 0–6 days was 173,853, and the number of deaths at 0–28 days was 231,283. Data for all age groups are summarized in Supplementary Table S3.

**Table 6:** Mortality for CBDs between 1990 and 2019 in China and globally stratified by age, expressed as numbers, rates, and trends.

Categories		1990		2019		1990–2019	*P*
		Mortality number (95%UI)	Mortality rate (95%UI)	Mortality number (95%UI)	Mortality rate (95%UI)	Mortality rate (AAPC 95%CI,%)	
China	0–6 days	30,563 (25,557, 37,087)	6,677.02 (5,583.31, 8,102.34)	7,253 (5,835, 9,243)	2,550.32 (2,051.62, 3,249.88)	−3.35 (−3.67, −3.02)	<0.001
	7–27 days	16,081 (12,548, 21,048)	1,179.52 (920.34, 1,543.82)	3,349 (2,750, 4,091)	392.65 (322.46, 479.61)	−3.74 (−4.02, −3.45)	<0.001
	0–28 days	46,644 (38,782, 57,674)	2,561.31 (2,129.59, 3,166.97)	10,602 (8,550, 13,183)	932.21 (751.82, 1,159.14)	−3.49 (−3.78, −3.19)	<0.001
Global	0–6 days	2,56,973 (2,08,743, 3,38,347)	9,837.58 (7,991.21, 12,952.80)	1,73,853 (1,44,240, 2,16,816)	6,759.20 (5,607.91, 8,429.58)	−1.30 (−1.37, −1.24)	<0.001
	7–27 days	1,11,522 (78,016, 1,62,720)	1,446.00 (1,011.56, 2,109.85)	57,431 (47,782, 71,397)	751.09 (624.91, 933.75)	−2.24 (−2.30, −2.19)	<0.001
	0–28 days	3,68,494 (2,90,856, 4,98,647)	3,529.11 (2,817.13, 4,829.72)	2,31,283 (1,92,527, 2,89,090)	2,263.41 (1,884.13, 2,829.13)	−1.56 (−1.63, −1.50)	<0.001

AAPC, average annual percentage changes; UI, uncertainty interval; CI, confidence interval.

## 4. Discussion

This study assessed trends in the incidence and burden of CBDs between 1990 and 2019 across China based on the GBD 2019. Findings showed an increase in the morbidity associated with CBDs in China. This was most apparent since 2013, likely due to the easing of the one-child policy (6). In 1979, China's total permanent population exceeded 962 million (21). The Chinese government introduced the one-child policy (13) as a population planning initiative. In 2013, with rapid economic development, the aging population, and the decline of the fertility rate (14), China eased its population policy, which eventually allowed all couples to have two children (15).

In 2016, the number of live births in China rose to 17.86 million, an increase of 7.9% since 2015 (22). After the two-child policy, births to women of advanced maternal age and those with maternal characteristics (e.g., diabetes mellitus) associated with birth defects increased (7, 12, 23–25). Accordingly, this study data show the age-standardized incidence rate for CBDs in China has been increasing since 2013, and the upward trend is predicted to continue between 2020 and 2030. It is recommended that the government and the health department improve access to quality maternal care for all pregnant women, including prenatal screening, vaccination, and vitamins and minerals, to prevent CBDs.

The present study showed the age-standardized incidence rate for CBDs between 1990 and 2019 across China was higher than the rate globally and in high- or middle-SDI regions, but lower than in low-SDI regions. Consistent with this, previous reports indicate the age-standardized incidence rate for CBDs is most severe in low-SDI regions of the world (4, 26). In China, in recent years, the age-standardized incidence rate for CBDs was slightly higher than the rate in low-SDI regions, possibly due to the introduction of the two-child policy. The higher age-standardized incidence rate for CBDs in China during this period may be due to its national conditions. Despite rapid economic development in recent years, China's development has been of low quality, and rural populations still make up the majority. Women in rural areas may be exposed to substances like pesticides and tobacco, and lack awareness of their harmful effects. Additionally, they are less likely to receive multivitamin supplements during pregnancy (27), unlike women in high- or middle-SDI regions who have better access to resources to address these hazards. According to the United Nations “*World Population Prospects 2022*” report (28), the world's population has reached 8 billion, and India is predicted to replace China as the world's most populous country in 2023. The age-standardized incidence rate for CBDs in India has been steadily increasing. Comparing China with India, which did not have a population planning initiative, confirms that the change in population policy in 2013 increased the incidence rate of CBDs in China. The preventative methods used by China to reduce CBDs may help other countries, such as India, strengthen national policies aimed at birth defect prevention and control.

This study data imply that male births were at higher risk of CBDs than female births between 1990 and 2019 in China. This may be due to the higher susceptibility of the Y chromosome than the X chromosome (29–31). Congenital heart anomalies were the leading type of CBD. In recent years, there was an alarming increase in congenital anomalies of the digestive system. This may be caused by genetic or environmental factors, with high mortality and poor prognosis. We recommend improving access to quality neonatal surgical care and strengthening the control of risk factors for congenital heart anomalies and congenital anomalies of the digestive system (32).

Globally, the under-five mortality rate has declined by 59% between 1990 and 2021 (https://data.unicef.org/topic/child-survival/under-five-mortality), but improving child survival in low-SDI regions remains an urgent concern. In China, the age-standardized mortality rate for CBDs decreased annually between 1990 and 2019, was lower than the rate globally and in middle-SDI regions, and is close to the level in high-SDI regions. Notably, in China, the age-standardized mortality rate for CBDs decreased after 2000. This progress is inextricably linked to the China Child Development Program (33), which was implemented in 2000. The China Child Development Program covers many aspects of policy, social security, health care, and education, and is updated every 10 years (34).

The present study indicates that CBDs were associated with high morbidity and low mortality in China between 1990 and 2019 compared with the trends in the global and high/middle/low-SDI, likely due to the market-oriented healthcare system, which has allowed medical institutions to become “self-sustaining”, and hospitals to offer “drug-based medical care” (35). Investment in preventative care is low, and most institutions are paid to treat rather than prevent disease, resulting in a lower mortality rate but no effective control of morbidity.

The mortality rate for CBDs between 1990 and 2019 in China was higher in males than females, with further research required to understand that difference. Congenital heart malformations were the leading cause of mortality from CBDs. Congenital heart malformations contribute to a significant disease burden globally (36), especially in China (37), warranting government investment in effective, accurate, and accessible medical services for diagnosis and treatment. Mortality associated with birth defects, in China and globally, occurred mainly in newborns, within one week of birth. Better management of newborns with CBDs is needed to improve the long-term survival of affected children.

In the past 30 years, China has had a high incidence rate of CBDs, causing a burden on families and society. However, the government has implemented policies and measures that have resulted in a significant decrease in CBDs deaths since 2000. Although the incidence rate may continue to increase due to two-child policies, medical technology advancements will control mortality rates. The government should continue to prioritize high-risk pregnant women and promote healthy habits (including healthy eating, regular sleep, appropriate exercise, and avoiding exposure to harmful substances) before conception to reduce fetal harm. Additionally, families with affected children should receive rehabilitation support to improve their quality of life.

### 4.1. Strengths and limitations

This study had several strengths. First, it provides comprehensive estimates of the trends and burden of CBDs at the national level in China. Second, China has a strict and well-developed birth registration and death reporting system for controlling the quantity and quality of the population, which guarantees the quality of the data. Last, the GBD2019 used standard methodology, which makes our estimates of the burden of CBDs in China comparable globally and with different SDI regions.

This study was associated with several limitations. First, the GBD 2019 had inherent limitations, which applied to this study. Second, data on incidence, mortality, and DALYs for CBDs across different provinces and economic regions, including urban and rural areas of China, were not available. Last, the dataset extends between 1990 and 2019, future studies with more recent data are warranted.

## 5. Conclusions

Morbidity associated with CBDs increased between 1990 and 2019 in China, accelerated by the adoption of the two-child policy, and ranked high globally. CBDs were more common in males than females, with congenital heart disease predominating. Mortality associated with birth defects occurred mainly in newborns, within one week of birth. These findings emphasize the need for prenatal screening and primary and secondary prevention strategies.

## Data Availability

Publicly available datasets were analyzed in this study. This data can be found here: http://ghdx.healthdata.org/gbd-results-tool.
