# Supplementary figures and images for: The burden of congenital birth defects between 1990 and 2019 in China: an observational study
Source: Front Pediatr. 2023 May 12;11:1170755. doi: 10.3389/fped.2023.1170755 (PMC10219611; doi:10.3389/fped.2023.1170755)

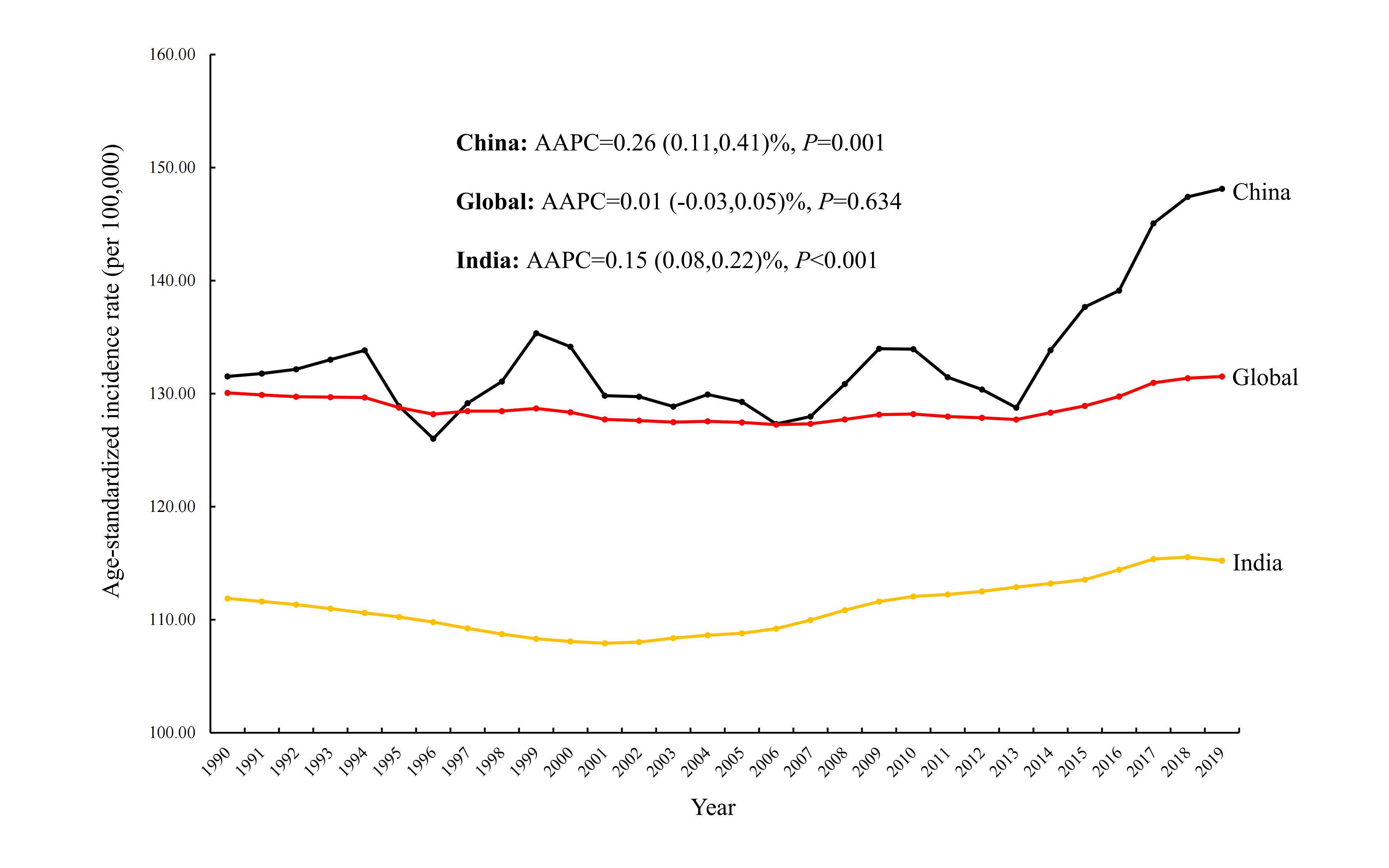

Supplement: Supplementary file 1 [file Datasheet1.zip › Figure S1.jpg]
